# Chain Formation during Hydrogen Loss and Reconstruction in Carbon Nanobelts

**DOI:** 10.3390/nano12122073

**Published:** 2022-06-16

**Authors:** Yuri Tanuma, Paul Dunk, Toru Maekawa, Chris P. Ewels

**Affiliations:** 1 Graduate School of Interdisciplinary New Science, Toyo University, Kujirai 2100, Kawagoe 350-8585, Japan; yuri.tanuma@eng.hokudai.ac.jp (Y.T.); maekawa@toyo.jp (T.M.); 2 Institut des Materiaux de Nantes Jean Rouxel (IMN), UMR6502 CNRS, Nantes University, 2 Rue de la Houssiniere, BP32229, 44322 Nantes, France; 3 Center for Advanced Research of Energy and Materials, Hokkaido University, Kita 13 Nishi 8, Kitaku, Sapporo 060-8628, Japan; 4 Ion Cyclotron Resonance Program, National High Magnetic Field Laboratory, Florida State University, Tallahassee, FL 32310, USA; dunk@magnet.fsu.edu; 5 Bio-Nano Electronics Research Centre, Toyo University, Kujirai 2100, Kawagoe 350-8585, Japan

**Keywords:** nanobelts, polycyclic aromatic hydrocarbons (PAH), cyclic polymers, hydrogen loss, fullerenes, carbon chains

## Abstract

Using laser-induced vaporisation to evaporate and ionise a source of curved polyaromatic hydrocarbons (carbon nanobelts), we show collision impacts between species cause mass loss and the resultant ions are catalogued via mass-spectrometry. These data are interpreted via a series of “in-silico”-simulated systematic hydrogen-loss studies using density functional theory modelling, sequentially removing hydrogen atoms using thermodynamic stability as a selection for subsequent dehydrogenation. Initial hydrogen loss results in the formation of carbyne chains and pentagon-chains while the nanobelt rings are maintained, giving rise to new circular strained dehydrobenzoannulene species. The chains subsequently break, releasing CH and C_2_. Alternative routes towards the formation of closed-cages (fullerenes) are identified but shown to be less stable than chain formation, and are not observed experimentally. The results provide important information on collision degradation routes of curved molecular carbon species, and notably serve as a useful guide to high-energy impact conditions observed in some astrochemical environments.

## 1. Introduction

Molecular carbon formation, transportation and transformation is one of the great scientific challenges in astrochemistry. Complex carbon molecules have been proposed as primary carbon sources in astrochemical environments such as the interstellar medium (ISM) [1], nebulae clouds [2,3] and supernovae remnants [4,5]. Carbon is expected to exist in a variety of forms including amorphous carbon and hydrocarbon dust [1] as well as in molecular structures. Of these, three key families are carbon chains [6], fullerenes [1,2,3,7], and polycyclic aromatic hydrocarbons (PAHs) [3,4]. UV-irradiation, hydrogen impacts and thermal cooling near supernovae present mechanisms for transformation between different species types. Growth and transformation models have predicted short chain presence during fullerene formation [8,9] while electron irradiation studies have demonstrated spontaneous transformation of planar aromatics into fullerenes [10], but precise carbonaceous transformation processes and pathways remain a subject of debate.

Curvature should play a critical role in carbonaceous species transformation and stability. As an example, localized curvature has been shown to aid hydrogen trapping in interstellar graphenic dust grain models, catalysing subsequent pairwise release giving H_2_ formation [11]. Curvature typically destabilises aromaticity and localises reactive sites [12]. It adds a driving mechanism for functionalisation and structural reorganisation in order to relieve curvature-induced strain, either by localising the curvature, or eliminating it through transformation from sp^2^- to sp^3^- bonded carbon. It is therefore interesting to examine the transformation behaviour under irradiation of curved aromatic hydrocarbons.

Despite the extensive available literature of damage-induced structural change in nanocarbons [10,13], it is very difficult to examine the damage breakdown process stepwise. This is because samples are typically non-uniform and data is either statistical across a range of related but different configurations, or based on observation of a single event (e.g., via high-resolution transmission electron microscopy, HRTEM). Here, we have chosen to study damage in carbon nanobelts [14] (CNBs, [(CH)_2_-C_6_H_2_]_2n_), part of a family of cyclic aromatic species that began with cycloparaphenylenes first synthesised in 2006 [15]. Nanobelts are of interest since they only contain hexagons and provide a continuous aromatic structure with uniform curvature. This is dependent purely on the ring circumference selected during synthesis. As such nanobelts can be considered archetypal curved polyaromatic hydrocarbons. At the same time, each molecule is identical to the others. This allows us to examine with statistical rigor the breakdown of identical, uniform nanorings, allowing us to identify the individual matter loss and transformation steps.

In this study we use laser-induced vaporisation to evaporate and ionise a source of carbon nanobelts. Collision impacts between species cause mass loss and the resultant ions are catalogued via mass-spectrometry. In order to understand the processes involved we perform a series of “in-silico”-simulated systematic hydrogen-loss studies using density functional theory (DFT) modelling. We sequentially remove hydrogen atoms using thermodynamic stability as a selection for subsequent dehydrogenation, allowing us to map and compare sequential hydrogen loss routes.

Our calculations show good agreement with experiment indicating initial pairwise hydrogen loss and local carbyne chain formation within the nanoring. The resultant species are a novel form of previously unreported circular strained dehydrobenzoannulenes, a new class of unsaturated chain-based hydrocarbons of great literature interest [16,17,18]. Subsequent damage results in release of the chain ends, leading to subsequent C_2_ and CH loss.

## 2. Method

Experimental gas phase studies of the carbon nanobelt C_48_H_24_ were performed with a laser cluster source using a 532 nm pulsed Nd:YAG laser system. The C_48_H_24_ sample is surface coated onto a quartz rod. A channel 2 mm in diameter and ~8.5 mm in length runs from a pulsed valve (10–70 psi backing pressure, 800 μs pulse width) into the region containing the rod to introduce helium. A second channel 2 mm in diameter was directed into the target area to admit the laser beam. Vaporization of the target rod is achieved by a single laser pulse fired from the Nd:YAG laser (5 ns, 5 mJ per pulse, ~1.5 mm beam diameter) in conjunction with the opening of the pulsed valve to admit He. A channel 4 mm in diameter and ~8.5 mm in length was located downstream from the target, aligned with the helium introduction channel to achieve confinement and clustering of the vapor produced. The gas then enters high vacuum and undergoes a free jet expansion.

The molecular species are isolated in the gas phase by use of the Sustained Off-Resonance Irradiation (SORI) technique [19] and then probed by collision-induced dissociation (CID) [20] to provide circumstantial structural information. This is achieved by accelerating the trapped ions in cyclotron motion, in the presence of Ar used as a collision gas. It is this step that causes molecular fracture as discussed further below. The cluster source is coupled directly to a Fourier Transform Ion Cyclotron (FT-ICR) Mass Spectrometer [21] whose very high resolution enables unequivocal identification of the stoichiometry of each and every ionic species produced, as the resulting spectra are completely resolved. The FT-ICR mass spectrometer is custom-built with ultra-high resolution, based on a 9.4 T, 155 mm bore diameter actively shielded superconducting magnet [22,23,24]. The cluster source was housed in a source chamber (1 × 10^−7^ torr) evacuated by a large diffusion pump (3000 L/s). Ions produced in the cluster source were transported to the ICR cell via three stages of differential pumping, each supplied with a turbomolecular pump to achieve ultrahigh vacuum (10^−10^ torr) in the ICR cell. After exiting the clustering region, the ions were skimmed into an octopole ion guide (175 V_p-p_, 1.8 MHz, 570 mm length) and immediately transferred to a second octopole for ion accumulation. Ions were confined radially in the accumulation octopole (240 V_p-p_, 2.8 MHz, 160 mm) by a time-varying electric field generated by a radiofrequency applied with 180° phase difference to adjacent rods and axially by the application of positive voltages to the end caps at the conductance limits at either side of the accumulation octopole. Further experimental details are available in Ref. [21], and for a review of SORI-CID techniques as applied to the activation of large ions, the reader is referred to Ref. [25].

We detect only the positive ion distribution created by the totality of all possible sequences of neutral–neutral, neutral–charged and charged–charged processes that result in positively charged species that emanate from the nozzle orifice. The experimental setup allows us to extract the resultant molecular fragments either before or after intermolecular collisions have been allowed to occur.

All calculations were carried out based on the density functional theory (DFT) calculations under the local density approximation (LDA) using the AIMPRO code [26,27,28]. Pseudo potentials given by Hartwigsen, Goedecker, and Hutter (HGH) [29] were used. Wavefunctions are handled using a basis set of Gaussian type orbitals [30,31], in which 38 (12) independent functions and used for carbon (hydrogen) atoms. Charge density is handled using plane waves with a cut-off of 300 Ha. Spin polarisation was used in systems with an odd number of electrons (µ_B_ = 1) and averaged for systems with an even number of electrons. Since only positively charged species are measured experimentally, all calculations performed here are in the +1 charge state. Such charging is handled by introducing a uniform homogenous compensating background charge to the cell. Electron temperature is handled using a Fermi function with temperature of 0.04 eV to aid convergence. All molecules are modelled in large hexagonal cells with over 20 Å until the next molecule to ensure no interaction and to minimize the density of the homogenous background charge. Vibrational modes are determined from numerical energy double derivatives (0.2 au atom shifts). Structures were geometrically optimised until system energy was converged to better than 10^−7^ Ha, and positions to within 10^−7^ atomic units. Enthalpies (in eV) are quoted for hydrogen removal via

ΔH = E(C_48_H_n_) − E(C_48_H_n−1_) − ^1^/_2_E(H_2_)

where E(n) is the total energy of species n in isolation. For the +1 charge state it is assumed that H_2_ always remains neutral, i.e., the positive charge is carried by the nanoring in each case. Note that this approach uses H_2_ as a reference state and does not necessarily imply hydrogen extraction as H_2_.

## 3. Results

**Figure 1:**
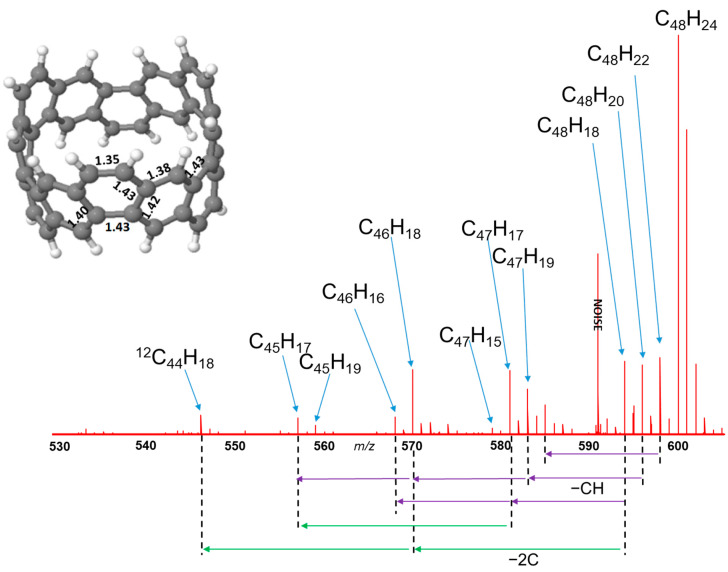
FT-ICR mass spectrum of C_48_H_24_ nanobelts showing dissociation through loss of varying quantities of H_2_, (CH) + H_2_ and (C_2_) + H_2_. *X*-axis indicates m/z ratio for positive ion species. Inset shows the DFT-optimised nanoring structure in + 1 charge state, calculated bond lengths marked in Angstroms.

The insert of Figure 1 shows the relaxed CNB (C_48_H_24_) structure. The ring has C_3_ symmetry, with significant variation in C-C bond-length around the ring. In the +1 charge state the exterior C-C bonds have strong local double bond character (1.35 Å) while the interior bonds become increasing aromatic (1.38–1.43 Å); the neutral charge state is similar with slightly dilated interior bonds (up to 1.45 Å). There are two hydrogen types by symmetry alternating around the ring in pairs: “exterior” hydrogen atom pairs are on neighbouring sites of the same hexagon, and “interior” hydrogen atom pairs on opposing sites of a given hexagon (1.09 Å bond length).

Fragmentation of the C_48_H_24_ nanobelt after collision-induced dissociation is shown in detail in Figure 1 (higher resolution details in Supplementary Materials Figure S1), showing clearly that the primary loss species come from removal of nH_2_, n = 1, …, 3. Similar intensity peaks correspond to removal of 3H_2_ + n_1_CH + n_2_C_2_, with (n_1_ + n_2_) ≤ 2, and increasingly weak signals for 2H_2_ + CH and H_2_ + CH loss. We are confident of the peak assignment since carbon isotopes can be readily distinguished due to the resolution of the spectrometer (Figure S1). Molecular dissociation occurs following intermolecular collisions, since the two mass spectrometry figures in Figure S2 show that exit species collected before collision do not show the presence of dissociated species in any notable amount.

**Figure 2:**
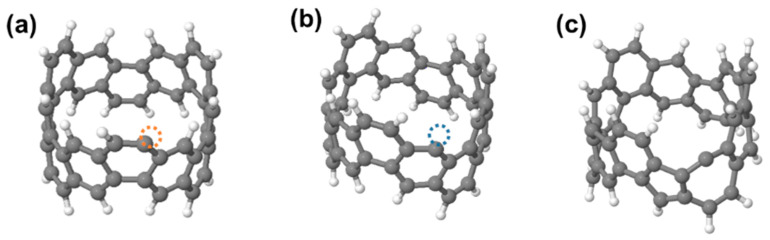
Molecular structures of C_48_H_23_, i.e., 1H-loss structures from an (**a**) exterior site and (**b**) interior site. Orange and blue dotted circles indicate dehydrogenated sites. (**c**) a pentagon-heptagon structure formed by Stone-Wales bond rotation of structure (**b**). Grey and white spheres indicate carbon and hydrogen atoms, respectively.

We now turn to simulations to examine potential dehydrogenation routes. Figure 2 shows three possible single dehydrogenation sites: exterior and “interior” hydrogenated sites (i.e., sites furthest and closest to the ring equator, respectively), and a third alternative where the structure in Figure 2b undergoes a 90° C-C bond rotation about the bond centre (a Stone-Wales (SW) bond rotation), for the non-circumferential bond of the dehydrogenated carbon atom. The result is that two hexagons are replaced with a pentagon-heptagon pair, with the dehydrogenated carbon sitting in the heptagon. Single hydrogen atom removal is highly endothermic (+3.49 eV for the interior site, +3.67 eV exterior and +4.21 eV for the bond rotated structure) due to the creation of a dangling carbon bond.

We next remove a second hydrogen atom from all possible symmetrically distinct sites of these three structures, testing this way nearly 30 2H-loss structures. Second hydrogen removal is always much easier than the first due to the electronic closed shell produced from pairwise dehydrogenation (the energetic cost for the second dehydrogenation step is typically less than half that of the first). This is consistent with a picture of pairwise dehydrogenation as seen experimentally in Figure 1.

**Figure 3:**
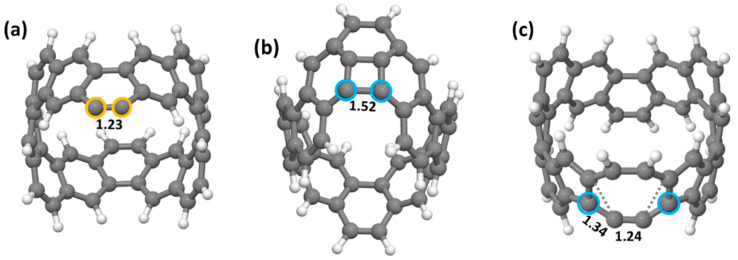
The most stable C_48_H_22_ structures (2H-loss) obtained by further hydrogen atom removal from the C_48_H_23_ structures in Figure 2 (Structures (**a**–**c**) correspond to Structures a–c in Figure 2 after removal of a second hydrogen atom). Values under bonds indicate the C-C bond lengths (Å). Orange and blue circles represent carbon atoms which have lost an external and internal hydrogen atom, respectively. Dotted lines indicate the original C-C bonds, which were rotated and reformed with the adjacent atoms (Stone-Wales bond rotations).

Three structures show significantly lower energy than all others (Figure 3). For the external dehydrogenation (Figure 2a), the most stable C_48_H_22_ loses a neighbouring pair of external hydrogen atoms (Figure 3a). The C-C bond between the dehydrogenated carbon neighbours stabilises the dangling bonds by rehybridising from a double (1.35 Å, Figure 1) to a geometrically distorted triple bond (1.23 Å, Figure 3a). This can be regarded as the shortest carbyne chain.

For interior dehydrogenation (Figure 2b), the most stable structure has the two closest internal hydrogen atoms removed. In this case, the resultant dangling bonds point towards each other and are removed by forming a new single bond and creating a square (Figure 3b). The three interior C-C bonds of the square remain at 1.41–1.42 Å, while the fourth bond, newly formed after dehydrogenation, is 1.52 Å, showing its single bond character. This rebonding creates a curved surface resembling locally the curved surface of a fullerene cage.

For the bond-rotated structure (Figure 2c), the lowest energy dehydrogenation process is the mirror of the first, i.e., removal of the nearest internal hydrogen followed by another bond rotation (SW) on the other side of the heptagon. The result is a conversion from three hexagons in the initial nanoring structure, to pentagon-heptagon-hexagon for the first dehydrogenation step, to pentagon-octagon-pentagon for the second. The two dehydrogenated carbon atoms form the exposed side of the octagon, rehybridising to a carbyne chain with bonds of 1.24 and 1.34 Å (Figure 3c).

Thus, we have three candidate structural routes for dehydrogenation resulting in either carbyne chains (Figure 3a), cage closing towards fullerene structures (Figure 3b) or carbyne chain formation linked with end pentagons (Figure 3c). For 2H-loss, this latter SW-carbyne route (c) is the least stable and the fullerene route (b) the most stable, although the energetic difference is very small (+0.03 eV/H atom).

**Figure 4:**
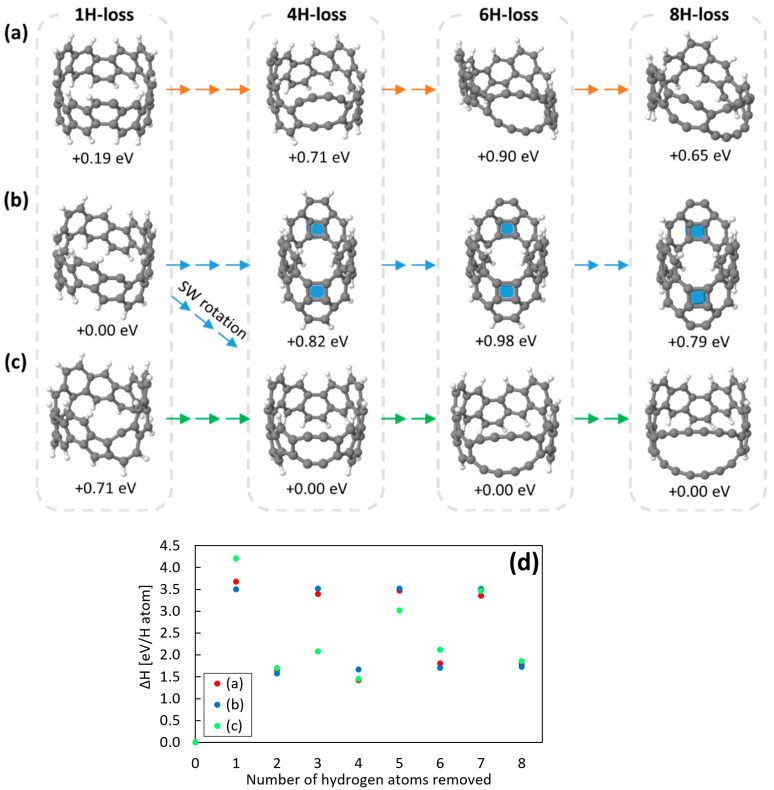
Carbon nanobelt maps (**a**–**c**) show key steps in the sequence of structures formed after sequential dehydrogenation. Route (**a**) begins with removal of exterior hydrogen atoms, route (**b**) with interior hydrogen atoms, and route (**c**) interior hydrogen atoms coupled to C-C bond rotations, as described in the text. The 1H-loss structures for (**a**–**c**) are shown in more detail in Figure 2. Grey and white spheres represent carbon and hydrogen atom, respectively. Each single arrow represents the removal of one hydrogen atom. Carbon squares are shaded in blue. The enthalpy value under each molecular model indicates the relative stability between the three isomers grouped vertically by grey dashed lines, i.e., between structures with the same number of hydrogen atoms (+0.00eV is the most stable structure each time). Importantly, the 4H-loss structure formed along the route (**c**) can also be formed from the 1H- and 2H-loss structures formed along (**b**) by the bond rotation. (**d**) shows the calculated enthalpy for removal of a single hydrogen atom from the previous structure for the three routes (**a**–**c**). A complete map showing every step is included in [App app1-nanomaterials-12-02073] Supplementary Materials Figure S3.

We now continue sequentially removing hydrogen atoms from each structure, at each step selecting the most stable structure for subsequent hydrogen removal. The full sequences are given in Figure S3, a summary with key steps in Figure 4.

For the “fullerene” closure route (Figure 2b), the next step involves the formation of a second square on the opposing side of the ring (Figure 4b). This localizes curvature at the two “ends” with squares, allowing the rest of the ring to flatten and increase aromaticity. However, thereafter there is no symmetrically obvious location for a third subsequent H_2_ removal via this route, and instead we see a diversity of possible structures. Notably external hydrogen removal (as via the orange route) is energetically almost identical to the formation of further squares along the sides of the ring. Since this route is energetically much less stable than the alternatives, we do not pursue it further.

**Figure 5:**
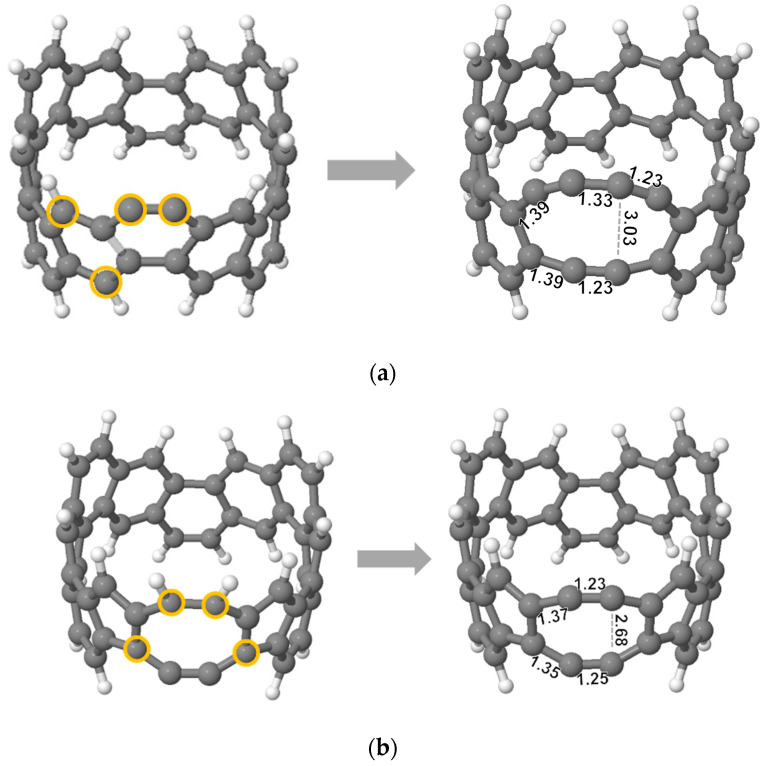
Further 2H loss from C_48_H_22_ to C_48_H_20_ (dehydrogenated carbon atoms circled in orange), for (**a**) the exterior route and (**b**) the SW-carbyne route. The bond grey-shaded in C_48_H_22_ breaks, creating two carbyne chains. Bond-lengths (Å) are symmetric about the two chain centres and for clarity are shown only once.

For the “carbyne” route starting with removal of two exterior hydrogen atoms (Figure 2a), for 4H it becomes more stable to break a cross-linking C-C bond, allowing the structure to rehybridise and open out into two carbyne chains (Figure 5a). While this process also results in strain relaxation in the ring with an increase in overall ring circumference, the two chains formed are not of equal length (containing two and four carbon atoms, respectively), which creates strong compression and tension, destabilising the structures. Thus, this route is also energetically relatively unfavourable. Bond length distribution shows a clear polyyne (single-triple) bonding in the shorter chain, with more cumulene-type bonding in the centre of the longer chain.

The third, SW-carbyne route (Figure 2c, Figure 3c and Figure 4c) results in significantly more stable structures than the other routes. Although these are also carbyne chains (Figure 4c), in this case the pentagon cross-bonding is maintained, the two carbyne chains are symmetric and contain only one C-C triply bound pair each (Figure 5b). In particular, this stabilises removal of the third hydrogen atom (Figure 4d), and as a result these structures are 0.7–0.8 eV more stable than the alternatives. The equivalent chain lengths avoid inter-chain strain while still allowing an increase in diameter, relieving curvature-induced strain in the rest of the ring. These structures form part of the family of strained dehydrobenzoannulenes [32], organic species with cross-linking triple-bonded chains.

We note furthermore that these routes are not mutually exclusive. As marked in Figure 4a, by rotating a C-C bond it is possible to shift from the “interior” fullerene route onto the SW-carbyne route. Thus, although the fullerene structures are energetically favoured for the initial hydrogen removal steps, they can easily divert to the more stable SW-carbyne structures upon further dehydrogenation.

As dehydrogenation continues (Figure 4), this route always results in the most stable structures, extending the two carbyne chains symmetrically. While this process can in principle continue around the ring, after removal of six hydrogen atoms, the carbyne chains are already very long (4 carbon atoms each). From this point on, the system becomes increasingly fragile and susceptible to breakage during intermolecular collisions. When the ends of the chains break away this allows the nanorings to open out and will not be reversible. Thereafter the unbonded ends of the carbyne chains will easily fragment during collision, losing C_2_ and CH species as seen experimentally (Figure 1).

We have calculated the vibrational modes associated with the SW-Carbyne chain structures (Figure 4c). With 2H removal (Figure 3c) a triple bond stretch mode appears at 2060.4 cm^−1^. This is slightly lower than the 2150 cm^−1^ mode associated with triple bonds in ion-implanted graphite [33] and is due to the local curvature and the +1 charge state in the current case, which will weaken the carbyne bonding and lead to frequency downshift. Long chain coupling, which can also downshift this mode, does not apply to the short carbyne chains in the current case [34]. In the 4H-loss case, this mode splits into two at 2068.3 and 2033.7 cm^−1^ due to the symmetry difference between the upper and lower chain, respectively (the lower lying more axially), showing weak vibronic coupling between the chains. In the 6H-loss case the chains are longer, the ring-induced curvature is less dominating and the +1 charge more distributed, and as a result the frequencies rise towards the conventional triple bond values, reaching 2080.8–2051.6 cm^−1^. For the simple 2H-loss structure in Figure 3a where no bond rotation has occurred, the triple bond is less linear, and its stretch frequency is only 2046 cm^−1^. These frequencies are all higher than those seen during damage-induced merging of double walled nanotube bundles at 1855 cm^−1^ ascribed to short linear carbon chains (3 to 7 atoms long) forming as bridging precursors to the merging of the tubes [35]. This is likely because short chain bonding will be highly sensitive to the nature of the end connections to the host nanocarbon.

There are no strongly localized characteristic modes on the pentagons at the chain ends, their modes instead strongly coupled to the rest of the structure. Two peaks at 1283/1302 cm^−1^ show relatively strong pentagon character, but as these lie very close to conventional damage peaks in carbon materials such as the D-peak in graphite they cannot serve as indicators for the presence of local hydrogenated pentagons.

## 4. Discussion and Conclusions

Experimental collision-induced mass loss of laser-ablated gas-phase C_48_H_24_ nanorings occurs through the loss of nH_2_, n = 1–3, followed by subsequent loss of nC_2_ and/or nCH, n = 0–2. Theoretical modelling of dehydrogenation routes, selecting favoured structures based on their relative enthalpic stability, provides a complete picture that is fully consistent with the experimental collision-induced dissociation mass spectrometry data. 

Initial pairwise hydrogen removal of up to six hydrogen atoms occurs through the formation of two parallel carbyne chains. In order to obtain parallel symmetric carbynes it is necessary to rotate carbon-carbon bonds at their ends, creating local pentagons that connect the chain pair to the rest of the nanoring. The carbyne chains stabilize the dangling bonds produced through dehydrogenation by rehybridising to triple bonds, with the added energetic advantage that the ring diameter can increase, relieving the internal curvature induced strain of the ring.

As dehydrogenation progresses and the chains become longer, intermolecular collisions break open the rings at the carbyne ends, and subsequent mass loss occurs through removal of C_2_ and CH from the chain ends. Alternative mass-loss routes, notably through introduction of further local curvature and closure towards fullerene-type structures, are energetically unfavourable beyond the first dehydrogenation step.

There are many literature examples of damage giving rise to both carbyne [36], and fullerene-like structures. Electron irradiation of small carbon flakes on graphene produces vacancies and results in closure to fullerenes [10], and metal templating also appears to catalyse fullerene formation during both growth and irradiation [8,21]. Carbyne formation is seen during irradiation damage of double-walled nanotube bundles [35] and graphite [33], and spontaneous short-lived chains are predicted to form during fullerene growth from small amorphous carbon clusters [9]. A common argument for carbyne production during irradiation is constrained thinning, where carbon is removed at a junction between nanoobjects, resulting in an increasingly thin connecting region which stabilizes as a carbyne chain before eventually breaking [37,38]. The current study is a limiting example of this; rather than thinning the connection between two carbon nanoobjects, there is instead thinning of the connection between two ends of the same curved nanoobject. Curvature in the aromatic network induced by the ring structure is thus a critical factor, applying tension to the damaged zone and thereby favouring carbyne formation. Additionally, the two hydrogenated nanoring edges in close proximity provide an easy route to rehybridisation into dual carbyne chains once dehydrogenated. Wider rings with more hexagons along their axis may become harder to rehybridise, although we note that field emission experiments from carbon nanotube tips suggested indirect evidence of carbon chain formation [39,40].

Finally, we note that these structures with parallel attached carbyne chains form part of the family of strained dehydrobenzoannulenes [32]. These molecules, of interest for their optoelectronic behaviour, are extremely difficult to synthesise through bottom-up organic chemistry routes, and the current “top-down” route of collision-induced dehydrogenation may be of interest as an alternative route to their production.

## Data Availability

The data presented in this study are available on request of the reader.

## Supplementary Materials

The following supporting information can be downloaded at: https://www.mdpi.com/article/10.3390/nano12122073/s1, Figure S1: Mass spectrum expansion of the dissociation spectrum of C_48_H_24_, demonstrating the extremely clear picture of products that are formed and detected. Notably the resolution allows us to distinguish between carbon isotope peaks and genuine hydrogen loss peaks, demonstrating that 2H-loss is strongly preferred over odd-number H-loss; Figure S2: FT-ICR mass spectra of laser desorbed C_48_H_24_ nanobelts, (A) SWIFT-isolated before collisional dissociation in the gas phase in an ultrahigh vacuum, and (B) after collision-induced dissociation. Fragments corresponding to loss of H-, C_2_ and CH- are clearly observable; Figure S3: Hydrogen-loss sequence under a charge +1 state started with a hydrogen bound to (a) “exterior” of a carbon edge, (b) “interior” of a carbon edge, and (c) “exterior” with additional C-C bond rotation to given terminating pentagons. Numbers in the coloured Schlegel diagrams (2D projections of the nanorings) represent the order of removed hydrogen atoms in each sequence. Values on arrows indicate energy difference (eV) between two structures. Dotted lines and zigzag lines in the Schlegel diagram show a new bond formed by removal of two adjacent hydrogen atoms and a broken bond by forming two carbon chains, respectively.
