# Supplementary material for: Chain Formation during Hydrogen Loss and Reconstruction in Carbon Nanobelts
Source: Nanomaterials (Basel). 2022 Jun 16;12(12):2073. doi: 10.3390/nano12122073 (PMC9230630; doi:10.3390/nano12122073)
Supplement: Supplementary file 1 [file nanomaterials-12-02073-s001.zip › nanomaterials-1747107-supplementary.pdf]

nanomaterials

MDPI

Supplementary Materials

Chain Formation during Hydrogen Loss and Reconstruction in Carbon Nanobelts

Yuri Tanuma [1,2,3], Paul Dunk [4], Toru Maekawa [1,5] and Chris P. Ewels [2,*]

[1] Graduate School of Interdisciplinary New Science, Toyo University, Kujirai 2100, Kawagoe 350-8585, Japan; yuri.tanuma@eng.hokudai.ac.jp (Y.T.); maekawa@toyo.jp (T.M.)
[2] Institut des Materiaux de Nantes Jean Rouxel (IMN), UMR6502 CNRS, Nantes University, 2 Rue de la Houssiniere, BP32229, 44322 Nantes, France
[3] Center for Advanced Research of Energy and Materials, Hokkaido University, Kita 13 Nishi 8, Kitaku, Sapporo 060-8628, Japan
[4] Ion Cyclotron Resonance Program, National High Magnetic Field Laboratory, Florida State University, Tallahassee, FL 32310, USA; dunk@magnet.fsu.edu
[5] Bio-Nano Electronics Research Centre, Toyo University, Kujirai 2100, Kawagoe 350-8585, Japan
* Correspondence: chris.ewels@cnrs-imn.fr

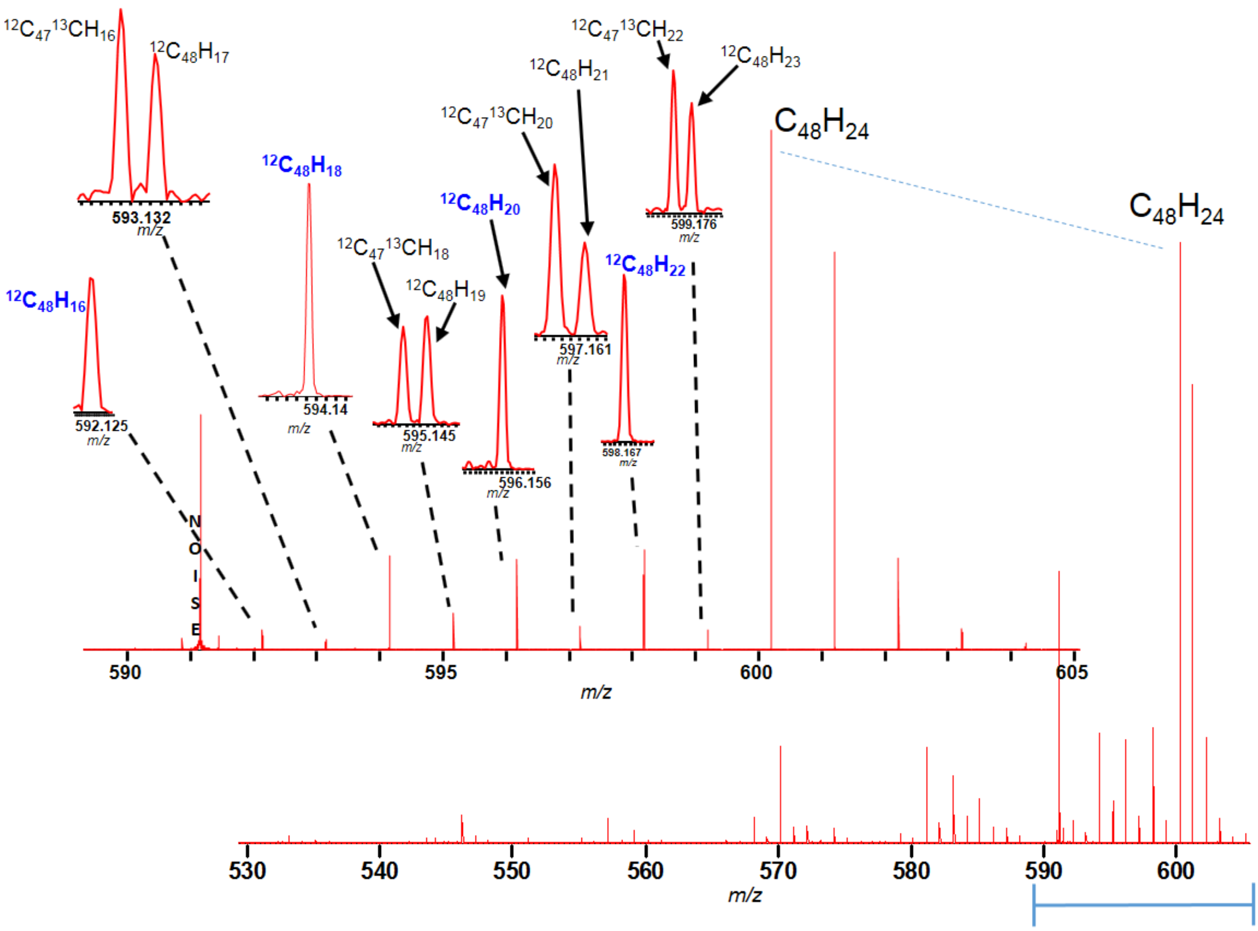

Figure S1. Mass spectrum expansion of the dissociation spectrum of $C_{48}H_{24}$, demonstrating the extremely clear picture of products that are formed and detected. Notably the resolution allows us to distinguish between carbon isotope peaks and genuine hydrogen loss peaks, demonstrating that 2H-loss is strongly preferred over odd-number H-loss.

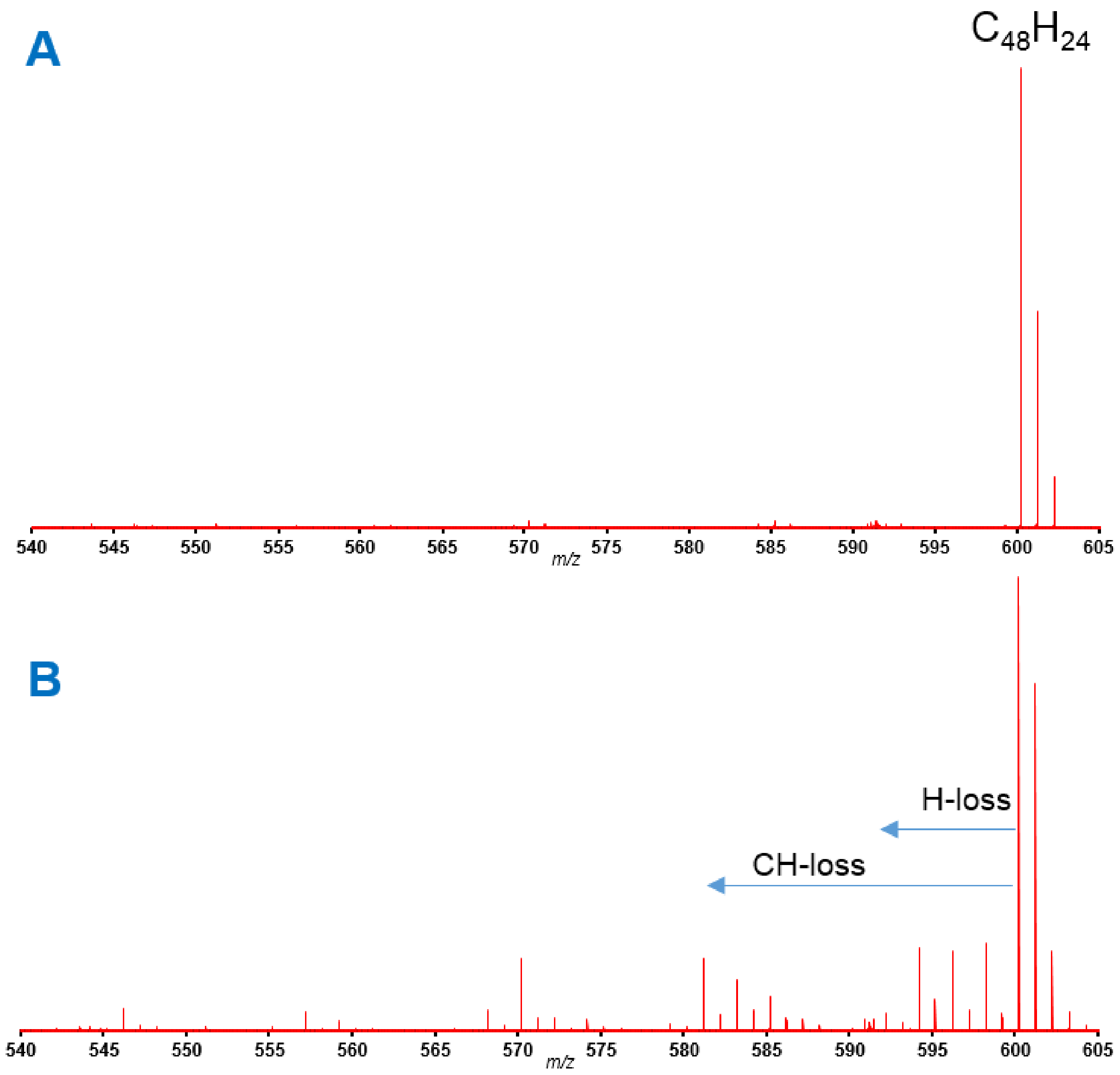

Figure S2. FT-ICR mass spectra of laser desorbed $C_{48}H_{24}$ nanobelts, (**A**) SWIFT-isolated before collisional dissociation in the gas phase in an ultrahigh vacuum, and (**B**) after collision-induced dissociation. Fragments corresponding to loss of H-, C_2 and CH- are clearly observable.

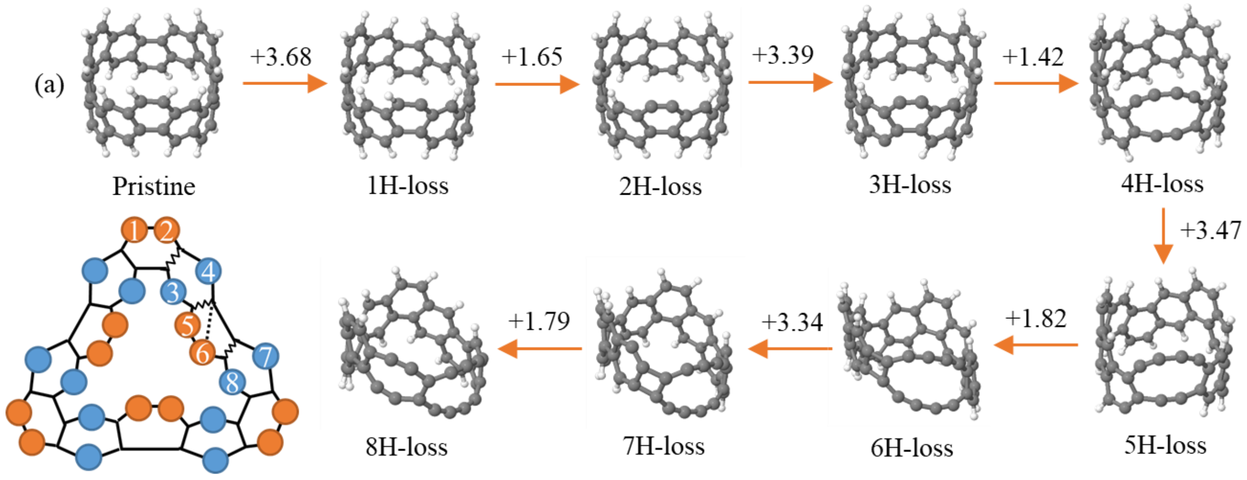

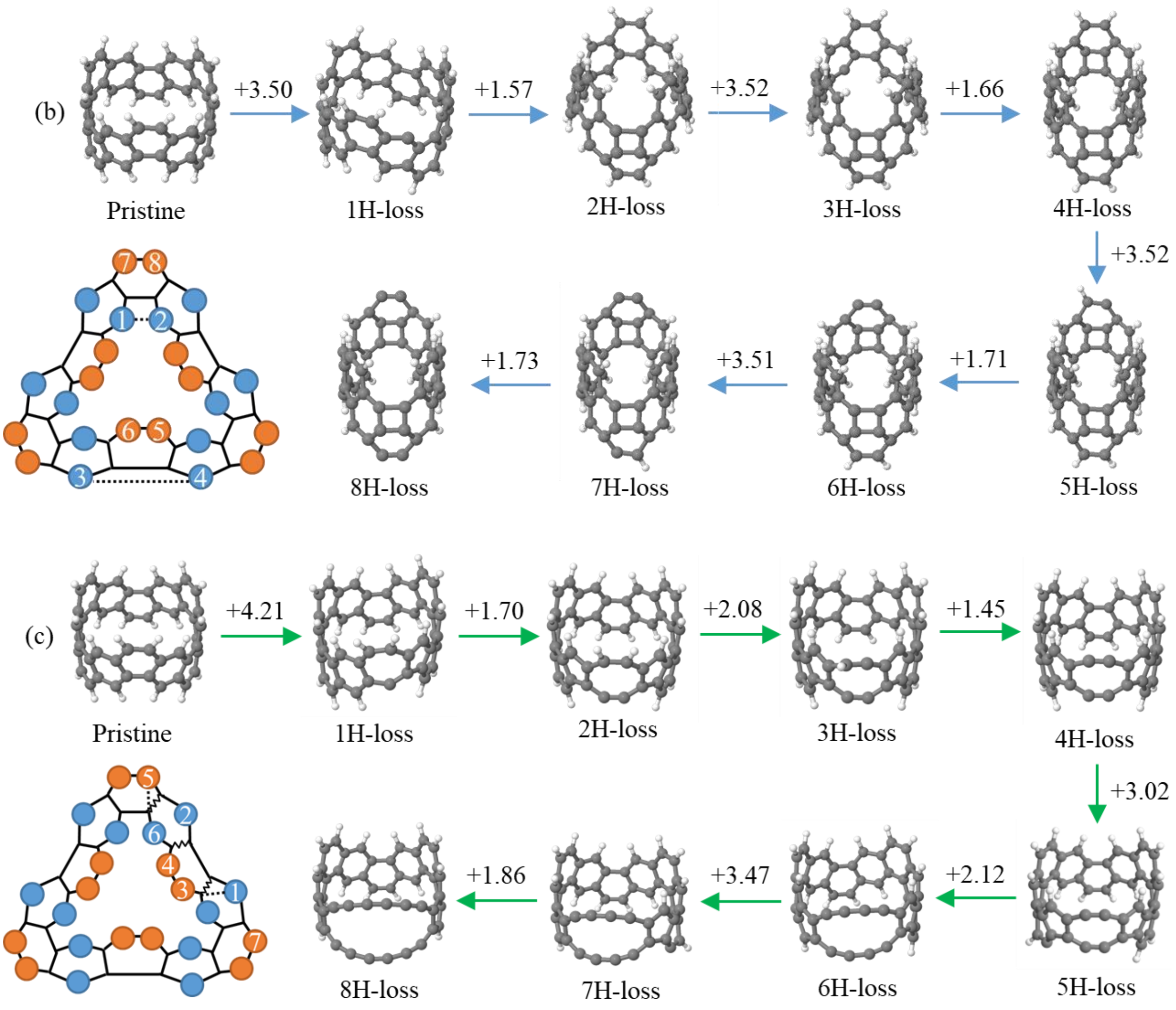

Figure S3. Hydrogen-loss sequence under a charge +1 state started with a hydrogen bound to (**a**) "exterior" of a carbon edge, (**b**) "interior" of a carbon edge, and (**c**) "exterior" with additional C-C bond rotation to given terminating pentagons. Numbers in the coloured Schlegel diagrams (2D projections of the nanorings) represent the order of removed hydrogen atoms in each sequence. Values on arrows indicate energy difference (eV) between two structures. Dotted lines and zigzag lines in the Schlegel diagram show a new bond formed by removal of two adjacent hydrogen atoms and a broken bond by forming two carbon chains, respectively.
